# The lack of *Tex44* causes severe subfertility with flagellar abnormalities in male mice

**DOI:** 10.1186/s11658-024-00587-5

**Published:** 2024-05-15

**Authors:** Sophie Dupuis, Marie-Sophie Girault, Morgane Le Beulze, Côme Ialy-Radio, Luis Bermúdez-Guzmán, Ahmed Ziyyat, Sandrine Barbaux

**Affiliations:** 1 Université de Paris, Institut Cochin, INSERM, CNRS, 75014 Paris, France; 2 Cancer Research UK Cambridge Institute, University of Cambridge, Cambridge, UK; 3 Service d’Histologie, d’Embryologie, Biologie de La Reproduction, AP-HP, Hôpital Cochin, 75014 Paris, France

**Keywords:** Spermatogenesis, Male infertility, Flagellar bending, Sperm, Mouse model

## Abstract

By analyzing a mouse Interspecific Recombinant Congenic Strain (IRCS), we previously identified a quantitative trait locus (QTL), called *Mafq1* on mouse chromosome 1, that is associated with male hypofertility and ultrastructural sperm abnormalities. Within this locus, we identified a new candidate gene that could be implicated in a reproductive phenotype: *Tex44* (*Testis-expressed protein 44*). We thus performed a CRISPR/Cas9-mediated complete deletion of this gene in mice in order to study its function. *Tex44*-KO males were severely hypofertile in vivo and in vitro due to a drastic reduction of sperm motility which itself resulted from important morphological sperm abnormalities. Namely, *Tex44*-KO sperm showed a disorganized junction between the midpiece and the principal piece of the flagellum, leading to a 180° flagellar bending in this region. In addition, the loss of some axonemal microtubule doublets and outer dense fibers in the flagellum’s principal piece has been observed. Our results suggest that, in mice, TEX44 is implicated in the correct set-up of the sperm flagellum during spermiogenesis and its absence leads to flagellar abnormalities and consequently to severe male hypofertility.

**Graphical Abstract:**

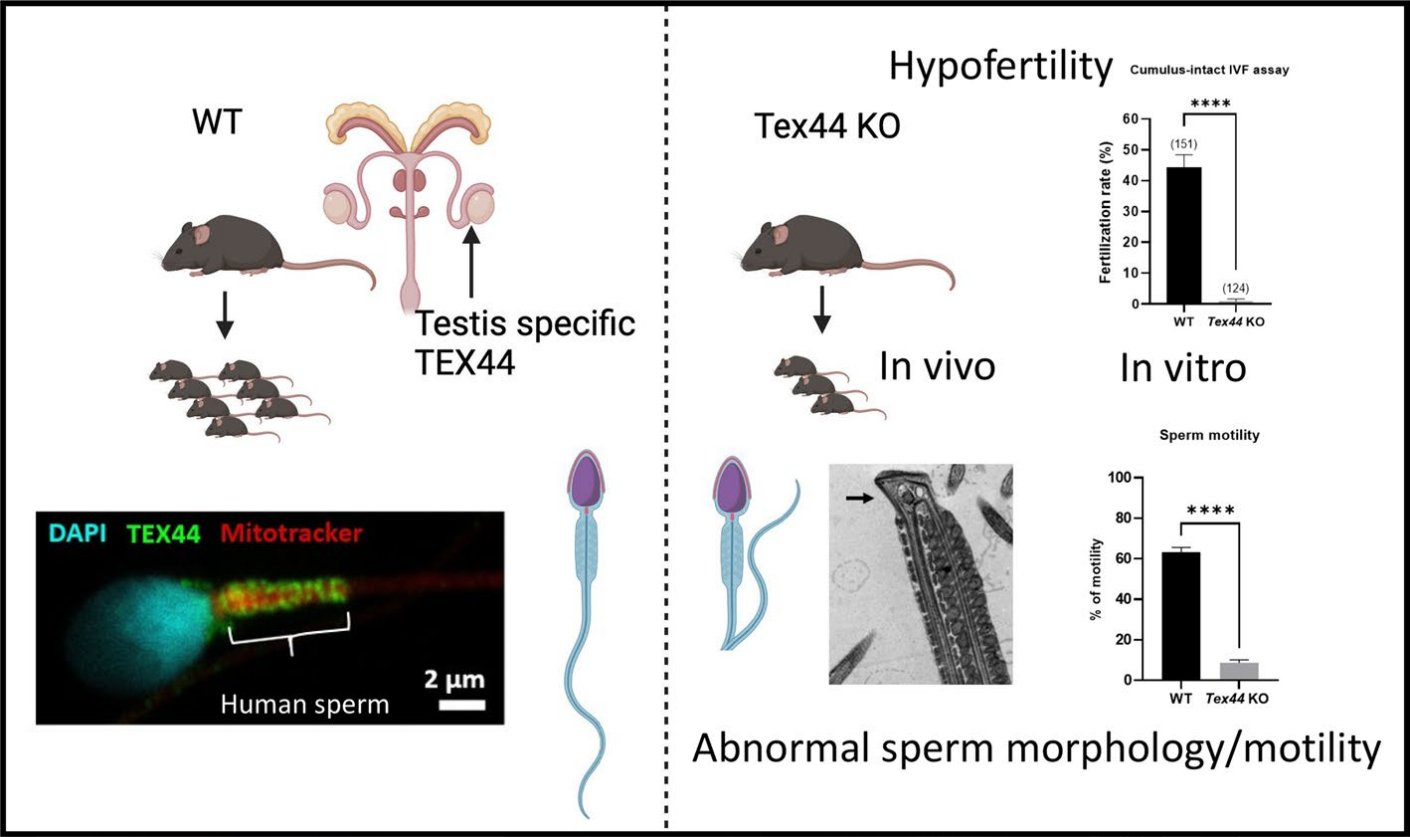

**Supplementary Information:**

The online version contains supplementary material available at 10.1186/s11658-024-00587-5.

## Background

Human infertility is a major public health issue that concerns 10–20% of couples of reproductive age. In half of the cases, the cause is due to male infertility [1, 2]. Amongst the different causes of male infertility, quantitative and/or qualitative sperm abnormalities can be involved such as low sperm count (oligozoospermia), reduced sperm motility (asthenozoospermia), and abnormal sperm morphology (teratozoospermia). Among the most severe forms of sperm flagellum defects responsible for male infertility, the Multiple Morphological Abnormalities of the Flagella (MMAF) syndrome, first described in 2014, characterizes patients presenting with a mosaic of anomalies including short, absent, coiled, bent, or irregular flagellum [3, 4].

Male infertility can be caused by genetic defects that affect spermatogenesis, a complex process in which sperm cells are produced in the testis. Genetic factors appear to have a strong share of involvement in human male infertility cases, with more than 120 genes implicated in the literature [5]. Still, most of infertility cases have an unidentified genetic cause [6].

Mouse models are very useful for exploring the function of candidate genes predicted to be involved in infertility since this functional investigation is difficult in humans. Thanks to the analysis of a specific hypofertile Interspecific Recombinant Congenic Strain (IRCS) mouse line previously described, we were able to define the *Mafq1* (male fertility chromosome 1) quantitative trait locus. The latter is an interval on mouse Chromosome 1 related to male hypofertility and sperm morphological abnormalities [7]. In this *Mafq1* locus, we previously identified *Spata3* as a candidate gene and we demonstrated that the deletion of this gene partially reproduced the phenotype of the hypofertile IRCS strain [8]. However, we recently found that a newly identified testis-specific gene, *Testis expressed protein 44* (*Tex44*), was reported in one of the latest versions of the mouse and human genomes and is present within the *Mafq1* locus. The *Spretus* version of TEX44 accumulates some variations compared to the C57BL/6 reference one that could be suspected to affect its structure and/or function (Supplementary Table 1).

No data are available concerning the function of the TEX44 protein mainly because it lacks any predictable functional domain. Indeed, the *TEX44* gene belongs to the TEX family only because of its expression pattern in the testis and not because of a specific function. To date, 69 *TEX* or *Tex* genes have been described in the human or mouse genomes [9]. In the mouse model, three genes have been related to subfertility (*Tex17, 18, 40*), and eight genes have been associated with infertility (*Tex11, 12, 14, 15, 19, 19.1, 38, 101*). Among them, three genes (*TEX11*, *TEX14,* and *TEX15*) have been linked to azoospermia and/or infertility in humans [5, 9].

Here, we identified TEX44 as a sperm flagellar protein. We analyzed in silico its evolution, its expression, its structure, and its potential protein interactions. Then PHENOMIN-ICS produced a mouse line in which the *Tex44* gene was deleted in order to understand the function of this gene. We observed that male mice lacking *Tex44* expression are drastically hypofertile in vivo and in vitro and that *Tex44*-KO sperm exhibit several ultrastructural anomalies.

## Methods

### Ethics statement

All animal experiments were performed in accordance with national guidelines for the care and use of laboratory animals. Authorizations were obtained from local (C2EA-34, Comité d’éthique en matière d’expérimentation animale Paris Descartes) and governmental ethical review committees via the APAFIS Application (Autorisation de projet utilisant des animaux à des fins scientifiques), Authorization APAFIS #14124–2017072510448522 v26, A. Ziyyat (2018–2023).

### Generation and breeding of transgenic mice

*Tex44* knock-out (KO) mice were established at the MCI/ICS (Mouse Clinical Institute—Institut Clinique de la Souris-, Illkirch, France; http://www.ics-mci.fr/en/) platform of the Institut de génétique et de la biologie moléculaire et cellulaire (IGBMC) in Illkirch-Graffenstaden, France. Two pairs of RNA guides, one targeting the 5’ UTR of *Tex44* (gR86: TCCCAACCACCATAAGTAGC and gR85, CAACCACCATAAGTAGCAGG), the other the 3’ UTR (gR85, ACAGTTACCTAGTGCAGGGT and gR82, GGGGACAGTTACCTAGTGCA) were selected and produced by in vitro synthesis. Guides and WT Cas9 (IDT, Leuven, Belgium) were electroporated into fertilized oocytes. The genetic background of these embryos was C57BL/6N and was maintained as such during all further crosses, though C57BL/6 J oocytes could also be used only in IVF assays that did not produce offspring. Embryos were transferred at the 1-cell stage into the oviducts of B6/CBA pseudopregnant females. F0 founders of interest were characterized by Sanger sequencing of the PCR fragment overlapping the deletion and three of them were bred to obtain germ line transmission. Subsequent genotyping of CRISPR edited founders was performed by PCR amplification on DNA extracted from tail biopsies (NucleoSpin® Tissue, Macherey–Nagel, Düren, Germany) using the GoTaq Flexi polymerase (Promega, Madison, WI, USA) under standard PCR conditions. Primers obtained from Eurogentec, Liege, Belgium are listed in Supplementary Table 2.

A PCR product of 264 bp was observed with primers F2 -R6 when the expected deletion was observed and checked by sequencing (Eurofins Genomics, Les Ulis, France).

### RT-PCR

RNAs were extracted from adult testis using a Trizol (Life Technologies, Saint Aubin, France) protocol. Two micrograms of total RNA were treated with DNase I (Promega, Charbonnières les bains, France) at room temperature, then retrotranscribed using the MMLV enzyme (Life Technologies) in the presence of random hexamers. PCR amplification was performed using the Go Taq Flexi (Promega) using the manufacturer’s recommendations.

### In vivo fertility and fertilization assay

Sexually mature (8 to 14 weeks) *Tex44*-KO homozygous and wild-type littermates were mated with C57BL/6 J females of 7–8 weeks. The numbers of pups and litters were recorded after 3 weeks of gestation whose beginning was attested by the presence of a vaginal plug after the overnight mating.

Wild-type C57BL/6 J female mice of 5–8 weeks (Janvier Labs, Le Genest-Saint-Isle, France) were injected with 5 IU of pregnant mare serum gonadotropin (PMSG). After 48 h, they were superovulated with 5 IU human chorionic gonadotropin (hCG) (Intervet, Beaucouzé, France) and then mated overnight with sexually mature WT or *Tex44*-KO males in order to evaluate their capacity to fertilize in vivo. The next day, females showing a vaginal plug were sacrificed by cervical dislocation and their oocytes retrieved. Oocytes were cleared of cumulus cells and directly mounted in Vectashield/DAPI (Vector laboratories, Burlingame, CA, USA) for observation under UV light (Nikon Eclipse E600 microscope) in a blinded manner. Oocytes showing at least one fluorescent decondensed sperm head within their cytoplasm were considered fertilized. According to this, the fertilization rate (FR) was evaluated.

### Mouse oocyte and sperm preparation for In Vitro fertilization

*Oocyte preparation* Wild-type C57BL/6 J female mice of 5–8 weeks (Janvier Labs, Le Genest-Saint-Isle, France) were superovulated with 5 IU of pregnant mare serum gonadotropin (PMSG) and then 48 h later with 5 IU human chorionic gonadotropin (hCG) (Intervet, Beaucouzé, France). The next day, about 13 h after hCG injection, animals were sacrificed by cervical dislocation. Cumulus oocyte complexes were collected by tearing the ampulla wall of the oviduct, placed in FertiCult medium (FertiPro N.V, Belgium) supplemented with 3% BSA (Sigma–Aldrich), and maintained at 37 °C under 5% CO_2_ in air under FertiCult Mineral Oil (FertiPro N.V, Belgium). When experiments were performed with zona-free oocytes, cumulus cells were first removed by a brief exposure to hyaluronidase IV-S (1 mg/ml, Sigma–Aldrich). Then, the zona pellucida was dissolved with acidic Tyrode’s solution (pH 2.5, Sigma–Aldrich) under visual monitoring. Zona-free eggs were rapidly washed in a FertiCult medium, BSA 3% and kept at 37 °C under 5% CO_2_ atmosphere for 2 to 3 h to recover their fertilization ability.

*Sperm preparation* Mouse spermatozoa were obtained from the cauda epididymis of WT or *Tex44*-KO male mice (8–14 weeks old) and capacitated at 37 °C under 5% CO_2_ for 90 min in a 500 μl drop of FertiCult medium supplemented with 3% BSA, under FertiCult Mineral Oil.

### In vitro fertilization

Cumulus-intact or zona-free eggs were inseminated with capacitated spermatozoa, at a final concentration of 10^6^/ml or 10^5^/ml respectively, for 3 h, in a 50 μl drop of FertiCult medium, 3% BSA kept at 37 °C, 5% CO_2_, under FertiCult Mineral Oil. At the end of incubation, oocytes were washed and directly mounted in Vectashield/DAPI (Vector laboratories, Burlingame, CA, USA) for a blinded observation under UV light (Nikon Eclipse E600 microscope). Oocytes showing at least one fluorescent decondensed sperm head within their cytoplasm were considered fertilized. According to this, the fertilization rate (FR) was evaluated. To assess the fertilization index (FI) in zona-free assay, the number of decondensed sperm heads per oocyte was recorded.

### Mouse sperm parameter analysis

Sperm motility was assessed by Computer-Assisted Semen Analysis (CASA) using the CEROS II apparatus (Hamilton Thorne, Beverly, MA, USA). Mouse spermatozoa samples were obtained from the cauda epididymis of WT or *Tex44*-KO mice (8 to 10 weeks old) and capacitated for 2 h in a 500 μL drop of FertiCult medium supplemented with 3% BSA, under mineral oil, at 37 °C under 5% CO_2_. The movements of at least 2000 sperm cells per sample were analyzed in 20 μm chambers (Leja Products B.V., NieuwVennep, Netherlands) with a Zeiss AX10 Lab. A1 microscope (10 × objective), using the HT CASAII software. The settings were as follows: acquisition rate, 60 Hz; number of frames, 45; minimum head brightness, 175; minimum tail brightness, 80; minimum head size, 10 μm2; minimum elongation gate, 1%; maximum elongation gate, 100%; objective magnification factor, 1.2. Though all sperm analyses were planned as blinded experiments to reduce potential biases, the structural anomalies of *Tex44*-KO sperm were inevitably recognizable.

### Histological analysis and mouse sperm preparation

Testes from adult WT and *Tex44*-KO males were collected and fixed overnight in 4% paraformaldehyde in PBS. Samples were dehydrated, embedded in paraffin and cut in serial 5 μm thick sections on a microtome. Testis sections were rehydrated before Hematoxylin–eosin (H&E) staining.

For Papanicolaou staining, sperm were retrieved from cauda epididymes in FertiCult medium and spread onto a Superfrost Plus slide (ThermoFischer Scientific). Sperm cells were fixed by incubation with paraformaldehyde 4% in PBS for 10 min and stained, following the Papanicolaou protocol (Hematoxylin, OG6, EA50). Briefly, slides were washed in 95% ethanol and dipped in Harris hematoxylin for 3 min for nucleus counterstaining, and washed, stained with OG-6 dye (RAL diagnostics 05.12013, Martillac, France) and with EA-50 (RAL diagnostics 05.12019). Then slides were dehydrated (95% ethanol absolute ethanol and xylene) and mounted with a permanent mounting medium.

For the analysis of the sperm morphology, testes and the three parts of the epididymis (caput/corpus/cauda) were dissected and spermatozoa were retrieved in M2 medium at 37 °C. Sperm samples were washed in PBS 1X- BSA1%, centrifuged at 600 g for 5 min and immediately fixed in 4% Paraformaldehyde (Electron Microscopy Sciences, PA, USA) in PBS-1% BSA for 5 min. A drop of sperm suspension was smeared on a slide, air-dried and mounted with Vectashield-DAPI. Detection was performed using a Nikon Eclipse E600 microscope. Images were digitally acquired with a camera (Coolpix 4500, Nikon, Champigny sur Marne, France).

### Mouse sperm immunofluorescence staining

Sperm suspensions were permeabilized during 10 min in PBS 1 × , triton 0.2%, and were then saturated for 1 h in PBS-BSA 1% with triton 0.02%, at RT. Then they were incubated for 1 h with a polyclonal anti-SEPTIN4 antibody (HPA021587, Sigma Aldrich) at 1:50 in PBS-BSA 1% with triton 0.02% at RT for 2 h and washed three times in PBS-BSA 1%. A secondary antibody (anti-Rabbit-Alexa Fluor 488 at 10 μg/ml, ThermoFischer Scientific) was used at RT during 1 h. After repeated washing with PBS-BSA 1%, a drop of sperm suspension was smeared on a slide, air-dried and mounted with the VECTASHIELD® PLUS Antifade Mounting Medium with DAPI medium (Eurobio Scientific, Les Ulis, France). Detection was performed using a Nikon Eclipse E600 microscope. Images were digitally acquired with a camera (Coolpix 4500, Nikon, Champigny sur Marne, France).

### Mitochondrial staining

Mouse spermatozoa were obtained from the cauda epididymis of WT or *Tex44*-KO male mice (8–14 weeks old) and capacitated at 37 °C under 5% CO_2_ for 90 min in a 500 μl drop of FertiCult medium supplemented with 3% BSA, under FertiCult Mineral Oil. Then, MitoTracker™ Orange CMTMRos (M7510, Thermofisher) was added to the medium at a concentration of 500 nM for 30 min in the incubator at 37 °C under 5% CO_2_. Sperm cells were then spread onto a Superfrost Plus slide (ThermoFischer Scientific). After drying in the dark, sperm cells were fixed by incubation with paraformaldehyde 4% in PBS for 10 min. Since this Mitotracker**™** is a probe that passively diffuses across the plasma membrane and accumulates in active mitochondria, the intensity of fluorescence reflects the mitochondrial activity. Detection of fluorescence was performed using a Nikon Eclipse E600 microscope and images were digitally acquired with a camera (Coolpix 4500, Nikon, Champigny sur Marne, France). The intensity of fluorescence has been normalized with that of the background fluorescence of each image.

### Human sperm immunostaining

Human sperm were donated by patients undergoing an assisted reproductive technology (ART) program for in vitro fertilization (IVF) in the assisted reproductive laboratory of Cochin’s Hospital (Paris, France). Spermatozoa were collected from excess fresh sperm derived from IVF attempts. The GERMETHEQUE Biobank site of PARIS-COCHIN (BB-0033-00081) provided 6 samples of sperm. GERMETHEQUE obtained consent from each patient to use their samples (CPP 2.15.27). The GERMETHEQUE pilotage committee approved the design of this study under the number 20220101. The Biobank has a declaration DC-2021-4820 and an authorization AC-2019-3487.

Classical and super-resolution STED immunofluorescence staining were made on these samples.

For the super-resolution STED immunofluorescence staining specifically, upon reception the human sperm samples were first washed in Ferticult medium supplemented with 3% BSA. Then they were incubated with MitoTracker™ DeepRed FM (M22426, Thermofisher) at a concentration of 500 nM for 30 min at 37 °C under 5% CO_2_. For classic and super-resolution STED fluorescence staining Samples were then fixed in 4% Paraformaldehyde (Electron Microscopy Sciences, PA, USA) in PBS 1X for 5 min followed by 3 washes with PBS-BSA 1%. Sperm suspensions were then permeabilized during 10 min in PBS 1 × , triton 0.2%, washed three times with PBS-BSA1% and then saturated for 30 min in PBS-BSA 3%.

Then for both classical and super-resolution STED immunofluorescence, sperm samples were incubated with a polyclonal anti-TEX44 antibody (HPA056433, Sigma Aldrich) at 1:40 (5 μg/ml) in PBS-BSA 1% or with an anti-Rabbit IgG isotype control (5 μg/ml) (Ref:02–6102, Invitrogen) overnight at 4 °C. The next day, samples were washed three times in PBS-BSA 1%. Secondary antibodies (anti-Rabbit-Alexa Fluor 488, ThermoFischer Scientific, at 10 μg/ml for the classical immunofluorescence and Abberior^®^ STAR 580 diluted at 1:1000 for the super-resolution STED immunofluorescence) were used at RT during 1 h. For the classic immunostaining, after repeated washing with PBS-BSA 1%, a drop of sperm suspension was smeared on a slide, air-dried and mounted with the VECTASHIELD^®^ PLUS Antifade Mounting Medium with DAPI medium (Eurobio Scientific, Les Ulis, France). Detection was performed using a Nikon Eclipse E600 microscope and images were digitally acquired with a camera (Coolpix 4500, Nikon, Champigny sur Marne, France).

For the super-resolution STED immunofluorescence, after repeated washing with PBS-BSA 1%, sperm samples were incubated with Hoechst (3 μg/ml) for 15 min then washed with PBS-BSA 1%. Then a drop of sperm suspension was smeared on a slide, air-dried and covered with the mounting medium Invitrogen™ ProLong™ Gold Antifade Mountant (Ref: P36934, Invitrogen). The slides were then sealed with the dental silicone Eco-sil speed (Ref: 476.410, Rotec). STED images have been acquired using an inverted SP8X STED WLL Leica confocal microscope (Leica Microsystems) at 100 × magnification (HC PL APO 1.4 oil), zoom factor 4x. We used a 775 nm pulsed laser to deplete both TEX44 and mitochondrial signals.

### Western blot

COS-7 cells were grown in DMEM medium supplemented with antibiotics and fetal calf serum (Invitrogen). They were transfected by electroporation on a 4D-Nucleofector apparatus (Lonza, Visp, Switzerland) with a construct containing the human TEX44 open reading frame cloned upstream of the EGFP sequence in the pEGFP-N1 vector (Clontech). After 48 h, cells were pelleted and protein extracts were obtained from lysis in a Laemmli buffer. Sperm pellets were extracted using a 2% SDS, PBS 1X solution supplemented with protease inhibitors. After denaturation, proteins were run on a precast Mini-Protean TGX gel (Bio-Rad, Marnes la Coquette, France), transferred to a nitrocellulose membrane and probed with the anti-TEX44 antibody.

### Electron microscopy

Mouse spermatozoa from males were prepared as described above (IVF) and fixed by incubation in PBS supplemented with 3% glutaraldehyde (Grade I, Sigma) for 2 h at room temperature. Samples were washed twice in PBS and post-fixed by incubation with 1% osmium tetroxide (Electron Microscopy Sciences). Then, they were dehydrated by immersion in a graded series of alcohol solutions and embedded in Epon resin (Polysciences Inc.,Warrington, PA, USA). Ultra-thin sections (90 nm) were cut with a Reichert Ultracut S ultramicrotome (Reichert-Jung AG, Wien, Austria) and then stained with uranyl acetate and lead citrate. Sections were analyzed with a JEOL 1011 microscope and digital images were acquired with a Gatan Erlangshen CCD camera and Digital Micrograph software. Characterization of the different phenotypes observed was performed through double blind analysis.

### Statistical analysis and study design

Results are expressed as mean ± SEM of at least three independent experiments. For statistical analysis, t-Tests were performed using GraphPad Prism version 9.00 for Windows (GraphPad Software, La Jolla California USA). Differences were considered statistically significant when p-value < 0.05. When data were not normally distributed, a Welch’s correction was applied. The experimental groups and the calculation of sample size were obtained using the Experimental Design Assistant (EDA; https://eda.nc3rs.org.uk).

## Results

### *TEX44* is only present in placental mammals and its expression is testis specific.

**Fig. 1:**
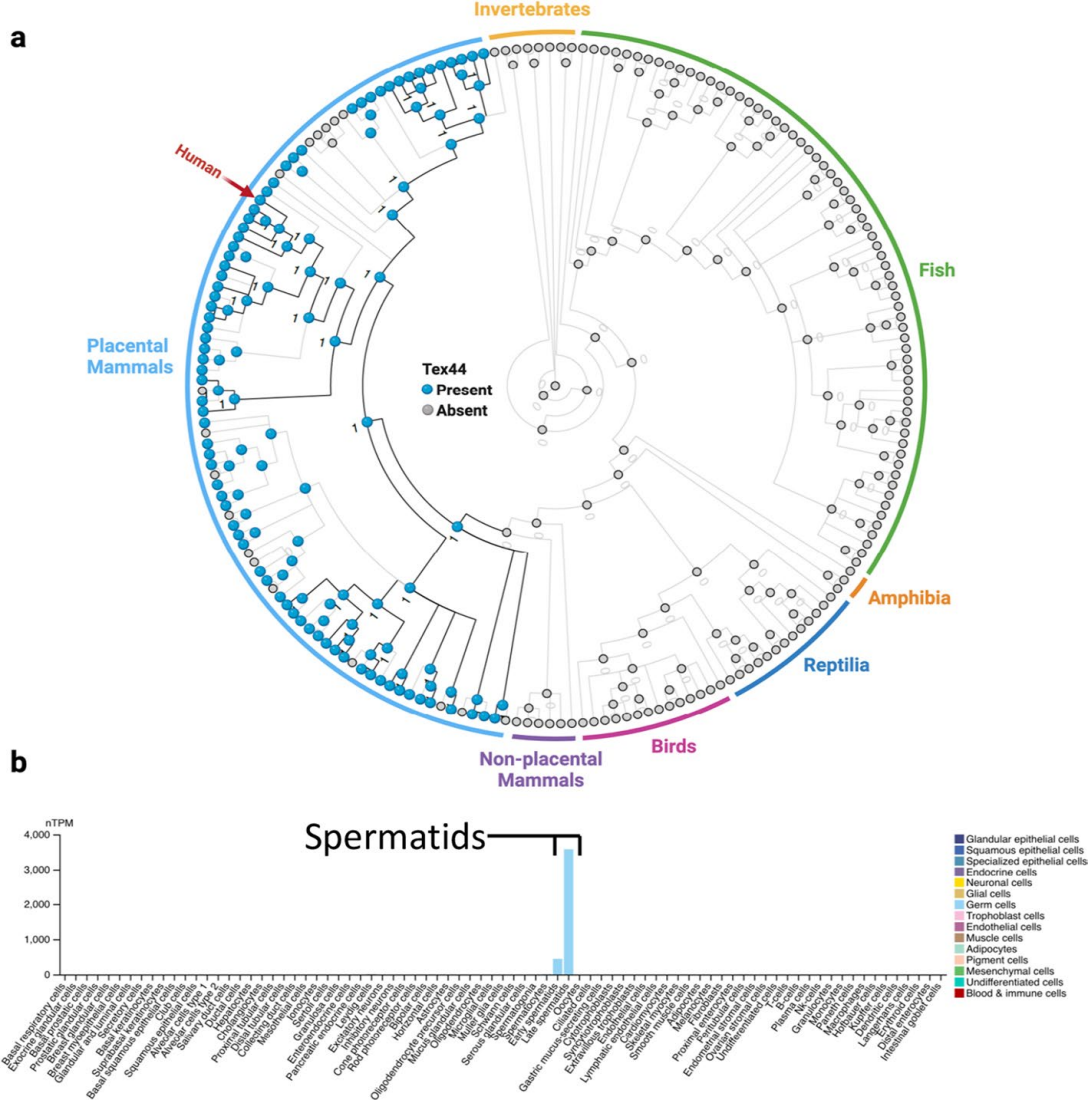
Evolution of *TEX44*. **a** The gene tree shows that *Tex44* first appeared in placental mammals (blue dots). Grey dots within placental mammals show instances in which there has been a gene loss of *TEX44*. **b** Single-cell RNA sequencing data confirmed the tissue-specific expression of *Tex44* in early and mainly late spermatids

To explore the evolutionary history of *TEX44*, we first used the Ensembl comparative genomics resources [10] (https://www.ensembl.org/index.html) to get the Gene Tree of *TEX44*. Interestingly, we noticed that the gene was only present in mammals, exclusively in Eutherians (placental mammals). To validate these findings, we used the human protein sequence of TEX44 to BLAST against the NCBI dataset (https://www.ncbi.nlm.nih.gov/) and we could not find homologous sequences in non-placental mammals and other vertebrates. Next, we analyzed the evolutionary pattern of gene gain/loss events, and we could find homologous sequences in the NCBI database for most of the placental mammals (Fig. 1a). Notably, protein sequence similarity between humans and other mammals was very low (mouse: 45.76% and ~ 80% for some primates; Supplementary Table 1), suggesting that this gene is evolving particularly fast in humans. The synteny between the *Mafq1* region on mouse chromosome 1 and the region on human chromosome 2 containing the *TEX44* gene and the common expression profile validate the orthologous status of the genes.

It has been reported that *TEX44* mRNA and protein are specifically expressed in the testis in both mice [9] and humans [11]. We used The Human Protein Atlas to interrogate this and found that based on single-cell RNA sequencing, *TEX44* is specifically expressed in early and mainly late spermatids (Tau specificity score = 0.98, where 1 means expression in a single cell/tissue type) (Fig. 1b). This has been also reported in mice [12, 13]. As *Spata3* is also exclusively expressed in testis, we also analyzed the evolutionary history of this gene and discovered that it is also present exclusively in placental mammals (Supplementary Fig. 1a). We confirmed that it is also specifically expressed in early and mainly late spermatids (Supplementary Fig. 1b), showing that both candidate genes from *Mafq1, Spata3* and *Tex44,* have a similar expression profile (Supplementary Fig. 1c).

### *Tex44*-KO male mice present drastic hypofertility in vivo and in vitro

**Fig. 2:**
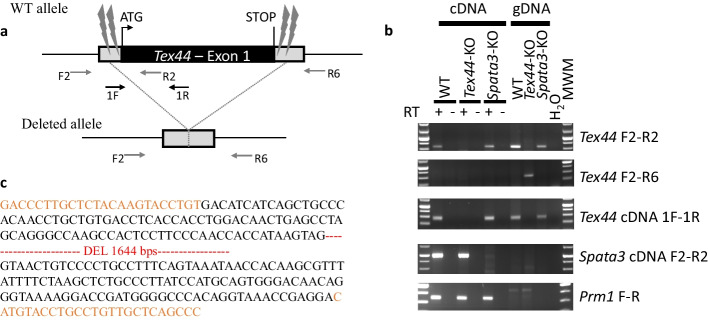
CRISPR/Cas9 strategy for *Tex44* mono-exonic gene deletion. **a** Map of the *Tex44* locus. RNA guides are represented upstream and downstream of the *Tex44* open reading frame as thunderbolts. The primers used for genotyping and cDNA amplification are represented by the F2, R2 and R6, and 1F, 1R arrows respectively. **b** Amplification of testis cDNA and genomic DNAs from WT, *Tex44*-KO and *Spata3*-KO samples for the *Tex44*, *Spata3* genes and *Prm1* as an expression control. MWM: molecular weight marker. **c** Sequence of the PCR product of the deleted allele, primers F2 and R6 are in orange

*Tex44* knock-out mice were generated using a CRISPR/Cas9 strategy at the Institut Clinique de la Souris (Celphedia, Phenomin, ICS, Illkirch). Guides located within both 5’ and 3’ UTR regions allowed the targeting and deletion of the complete open reading frame included in the unique exon of *Tex44*. Animals presenting a deletion of *Tex44* were validated by PCR amplification and sequencing of the locus as illustrated in Fig. 2. Animals were crossed over at least 4 generations and obtained in mendelian proportions to finally obtain homozygous knock-out animals (*Tex44*-KO). Though no specific antibody to reveal the absence of the TEX44 protein was available, the lack of *Tex44* mRNA could be validated in testis samples by RT-PCR (Fig. 2b). This additional internal PCR also performed on genomic DNA confirmed the complete absence of the *Tex44* gene.

**Fig. 3:**
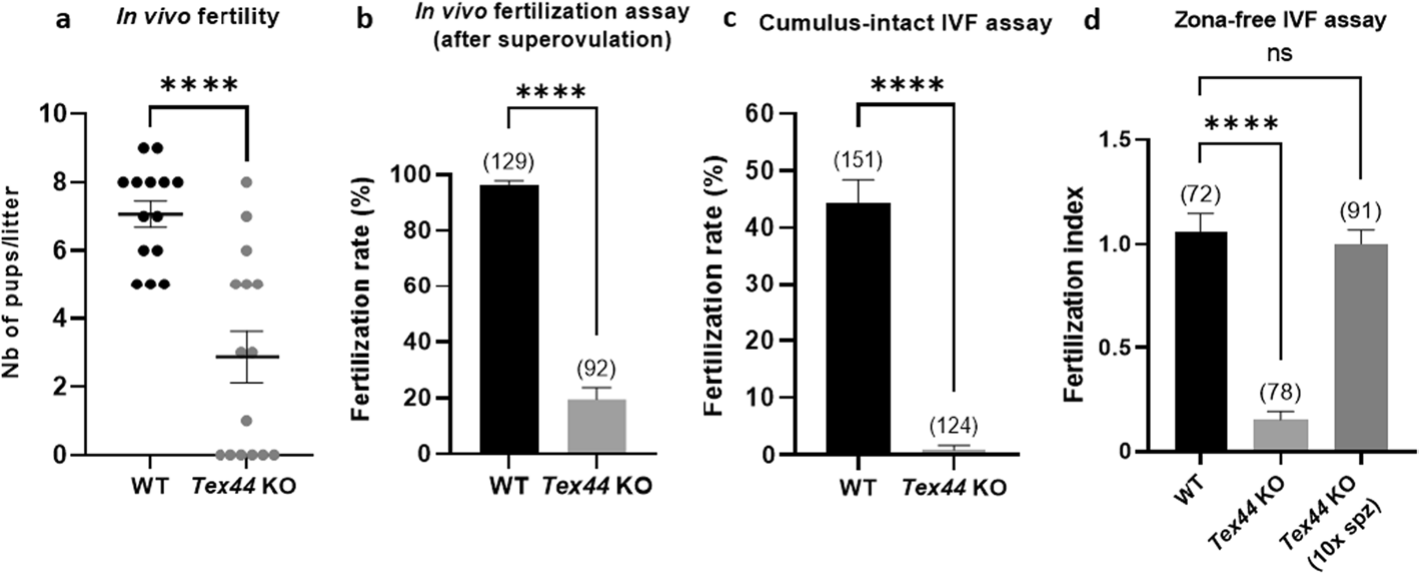
Evaluation of *Tex44*-KO male fertility in vivo (**a**, **b**) and in vitro (**c, d**). **a** WT females were mated with WT or KO males and litter size (mean ± SEM) was counted after 3 weeks of gestation whose beginning was attested by the presence of a vaginal plug after the overnight mating. **b** Superovulated WT females were mated with WT or KO males to evaluate their capacity to fertilize in vivo. The day following the overnight mating, only females showing a vaginal plug attesting that a mating had occurred were used for the experiments. The fertilization rate (FR) (mean ± SEM) was assessed by the in vivo fertilization assay. *Tex44*-KO males’ in vivo fertility was significantly decreased. **c** The fertilization rate (FR) (mean ± SEM) was assessed by cumulus-intact In Vitro Fertilization (IVF) assay at 10^6^ (WT and KO) sperm per ml. No fertilization was obtained with *Tex44*-KO sperm. **d** The fertilization index (FI) (mean ± SEM) was assessed by zona-free IVF assay at 10^5^ (WT or KO) or 10^6^ KO sperm per ml (10 times more concentrated). FI was significantly decreased when oocytes were inseminated with KO sperm compared to WT sperm. The FI was restored to WT values when the concentration of KO sperm was increased by 10 times. All experiments were repeated at least three times. **** *p* < 0.0001. Numbers between brackets indicate the number of oocytes

In order to test in vivo fertility, Wild-Type (WT) and *Tex44*-KO littermate males were mated with C57BL/6 J WT females. The average litter size was significantly different (*p* < 0.0001) when pups were born from WT males (7.1 ± 0.38; n = 14) compared to those born from *Tex44*-KO males (2.9 ± 0.75; *n* = 15) (Fig. 3a). When females showed a vaginal plug after mating but not followed by births, the number of pups per litter was recorded as null. Offspring of *Tex44*-KO males, though rare, were viable and normal. Homozygous females could breed normally.

In vivo fertilization assays were performed with superovulated C57BL/6 J WT females that were mated overnight with WT or *Tex44*-KO males. The next day, females showing a vaginal plug were sacrificed and cumulus-intact oocytes were retrieved to evaluate the in vivo fertilization rate (FR: the average number of fertilized oocytes among the total number of oocytes). The FR was significantly different: 96.1 ± 0.02% (*n* = 129) for the control condition (WT) and 19.6 ± 0.04% (*n* = 92) for the KO condition (*p* < 0.0001) (Fig. 3b).

For in vitro fertilization assays, cumulus-intact and zona-free oocytes from WT females were incubated with sperm from WT or KO males. For the cumulus-intact IVF assays, the FR was 44.4 ± 0.04% (*n* = 151) for the control condition (WT) and 0.8 ± 0.01% (*n* = 124) for the KO condition (*p* < 0.0001) (Fig. 3c). For the zona-free assays, the fertilization index (FI: the average number of fused sperm per oocyte) was 1.06 ± 0.09 (*n* = 72) for the control condition and 0.15 ± 0.04 (*n* = 78) for the KO condition (*p* < 0.0001). Interestingly, we were able to bring back the FI to control values in the KO condition by inseminating the oocytes with 10 times more sperm (FI = 1 ± 0.07, *n* = 91) (Fig. 3d).

These observations suggested that the deletion of *Tex44* drastically affects the successful process of fertilization both in vivo and in vitro.

### *Tex44*-KO male mice present sperm morphological abnormalities

**Fig. 4:**
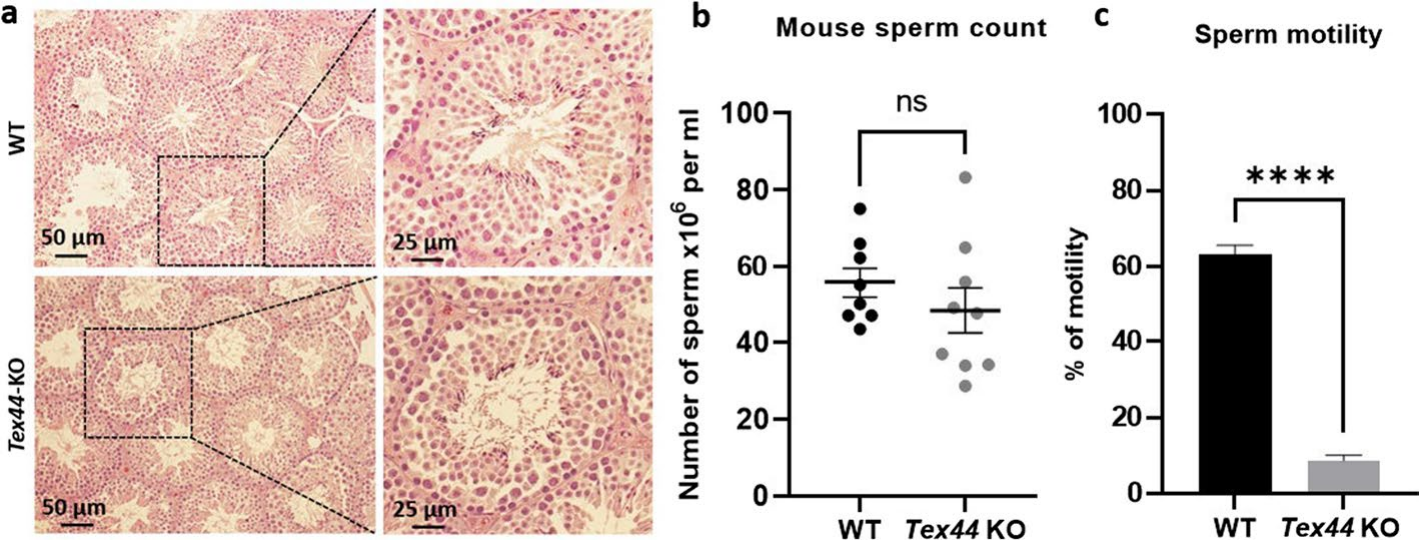
Histology of testes, sperm counts and motility analysis of WT and *Tex44*-KO males. **a** No obvious abnormalities of testes histology were observed between WT and *Tex44*-KO. **b** No significant difference was observed between WT and *Tex44*-KO cauda epididymal sperm counts. **c** Analysis of sperm motility by Computer Assisted Semen Analysis (CASA). CASA analyses showed a drastic reduction of motility of the *Tex44*-KO sperm compared to the WT sperm. Data are represented as the mean ± SEM of at least three sperm samples of each group of males (WT and KO). **** *p* < 0.0001

In order to better understand why *Tex44*-KO males are highly hypofertile in vivo and in vitro, we further characterized their testis and sperm cells. Histology of the *Tex44*-KO male's testes showed no obvious abnormalities (Fig. 4a) and the sperm counts from the epididymal cauda were not statistically different between WT and *Tex44*-KO males (55.7 ± 3.9 × 10^6^ sperm/ml for the WT males (n = 8) vs. 48.3 ± 5.8 × 10^6^ sperm/ml (*n* = 9) for the *Tex44*-KO males) (Fig. 4b). However, the Computer Aided Sperm Analysis (CASA) system revealed a drastic difference in sperm motility between WT sperm (63.2 ± 0.02% of motility) and *Tex44*-KO sperm (8.7 ± 0.01% of motility) (Fig. 4c).

**Fig. 5:**
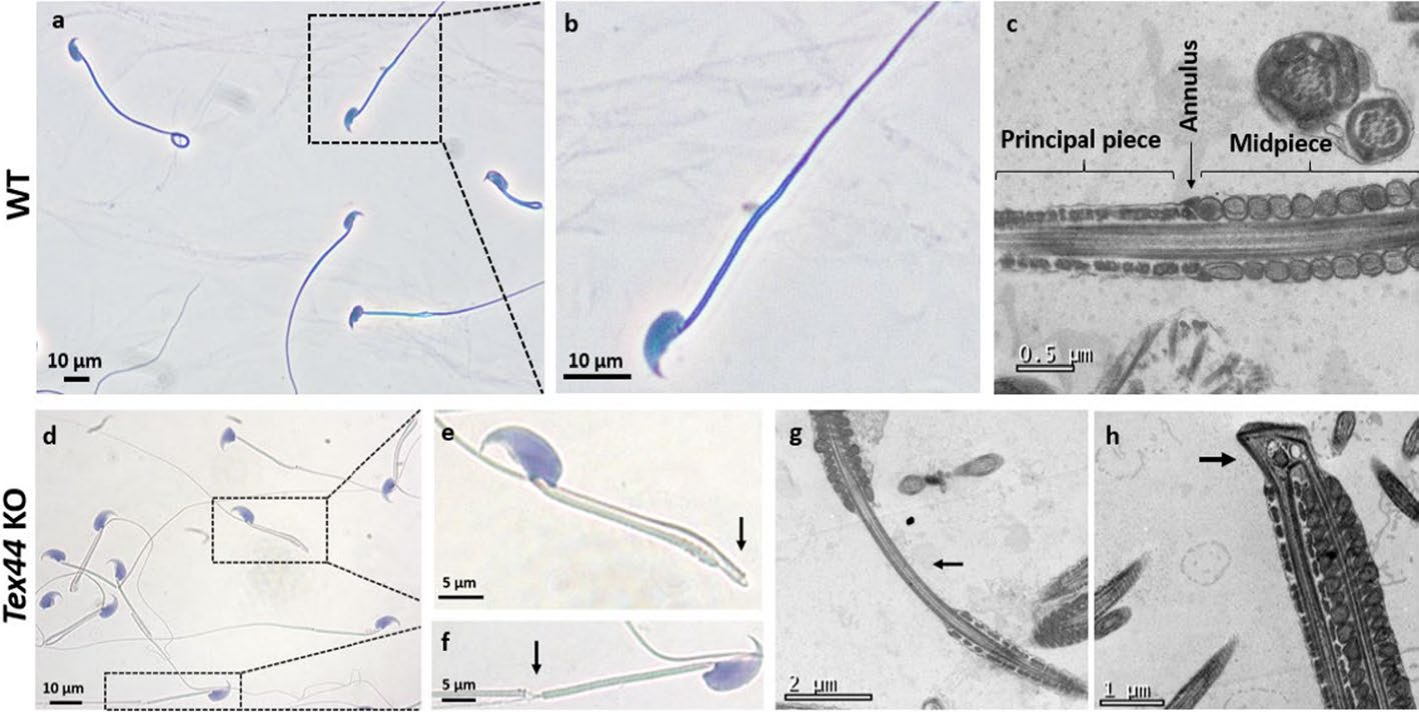
Characterization of the sperm morphology by light (**a**, **b**, **d**-**f**) and electron microscopy (**c**, **g**, **h**). Papanicolaou staining of WT and *Tex44*-KO sperm, retrieved from the cauda epididymis (**a**, **d**), with the associated enlargement of the framed areas respectively (**b**) for the WT and (**e**) and (**f**) for the *Tex44*-KO sperm. Example of a severe bending of the flagellum between the midpiece (MP) and the principal piece (PP), observed in *Tex44*-KO sperm in light microscopy (**e**) and electron microscopy (**h**) in comparison with WT sperm (**b**) and (**c**), respectively). Example of a MP-PP disjunction of the flagellum observed in *Tex44*-KO sperm in light microscopy (**f**) and electron microscopy (**g**). The arrows (**e**, **f**, **g**, **h**) show the two kinds of abnormalities observed on the sperm flagellum

Observation of sperm morphology under light microscopy showed obvious defects on the *Tex44*-KO sperm retrieved from the cauda of the epididymis (Fig. 5). These KO sperm showed disjunction (Fig. 5d,f) and severe hairpin bending (Fig. 5d,e) between the midpiece and the principal piece (MP-PP) compared to WT (Fig. 5a,b).

**Fig. 6:**
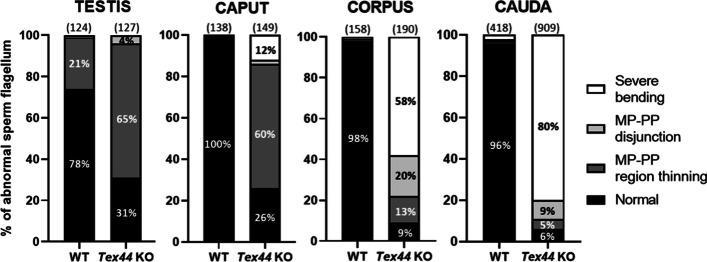
Comparison of the sperm flagellum abnormalities of WT and *Tex44*-KO sperm from the testis and from caput, corpus and cauda epididymis. While progressing through the different stages of maturation in the epididymis, the Tex44-KO sperm morphological phenotype gets worse, evolving from a majority of midpiece-principal piece (MP-PP) disjunction to a majority of severe bending of the flagellum. Numbers between brackets indicate the number of individual sperm observed

In order to stage these abnormalities observed on the flagellum, we isolated sperm from the testis and the three main regions of the epididymis (caput/corpus/cauda). The proportion of severe hairpin bending in the *Tex44*-KO sperm increased along the progression through the three regions of the epididymis. The frequency of this anomaly went from 12% in the caput to 58% in the corpus and finally 80% in the cauda (Fig. 6). No severe bending was observed in the testis sperm but 65% of these testicular *Tex44*-KO sperm showed a thinning between the midpiece and the principal piece.

### *Tex44*-KO male mice present other sperm ultrastructural abnormalities

**Fig. 7:**
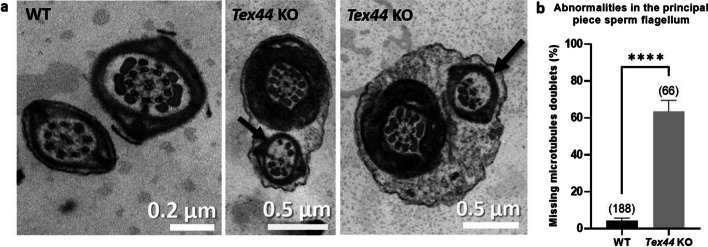
Electron microscopy analysis of cross-sections of WT and *Tex44*-KO sperm’s flagellum. **a** Examples of the lack of some microtubule doublets and their associated outer dense fibers in the principal piece of the *Tex44*-KO sperm’s flagellum. **b** Counting of sperm displaying the phenotype showed in panel (**a**), in WT and *Tex44*-KO cross-sections of the sperm’s flagellum. Numbers between brackets indicate the number of sperm flagellum’s cross-sections analyzed. **** *p* < 0.0001

Sperm samples were then analyzed by electron microscopy to confirm the ultrastructural defects observed previously and to reveal those that could not be visible through light microscopy. In addition to the sperm flagellar MP-PP disjunction and hairpin bending shown in Fig. 5g, h, transversal sections of the principal piece (PP) often showed the lack of some microtubule doublets and their associated outer dense fibers (4.2 ± 0.01% of PP abnormalities for the WT sperm *vs* 63.6 ± 0.06% for the *Tex44*-KO sperm). (Fig. 7a, b).

### Presence of the annulus in *Tex44*-KO male mice sperm

**Fig. 8:**
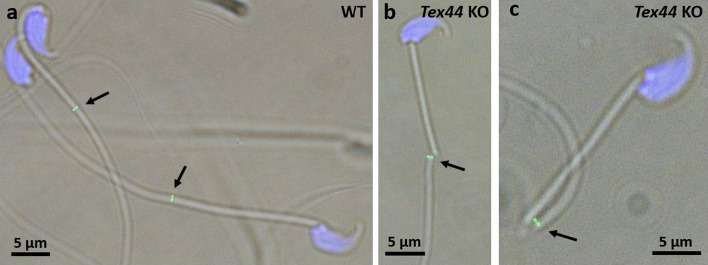
Presence of the annulus on the *Tex44*-KO flagellum. Immunostaining of WT (**a**) and of *Tex44*-KO (**b**, **c**) cauda epididymal sperm with an anti-SEPT4 antibody showed both a specific ring staining between the midpiece and the principal piece of the flagellum, despite the abnormal flagellum structure in the KO. Nuclei are counterstained in blue by DAPI

Given that *Tex44*-KO sperm exhibit flagellar disjunction and a hairpin bending at the junction between the MP and PP, we also interrogated the integrity of the annulus, a septin-based fibrous ring-like structure located between the MP and the PP of the flagellum. We thus performed immunofluorescence on cauda epididymal sperm with an anti-SEPT4 antibody to detect this conserved annulus protein [14]. A specific ring staining was observed between the MP and the PP of the flagellum in the WT and *Tex44*-KO sperm despite their abnormal structure in this latter (Fig. 8). This result suggests that the annulus structure does not seem to be impacted by the absence of TEX44 in the sperm.

### Lower mitochondrial activity of the *Tex44*-KO sperm

**Fig. 9:**
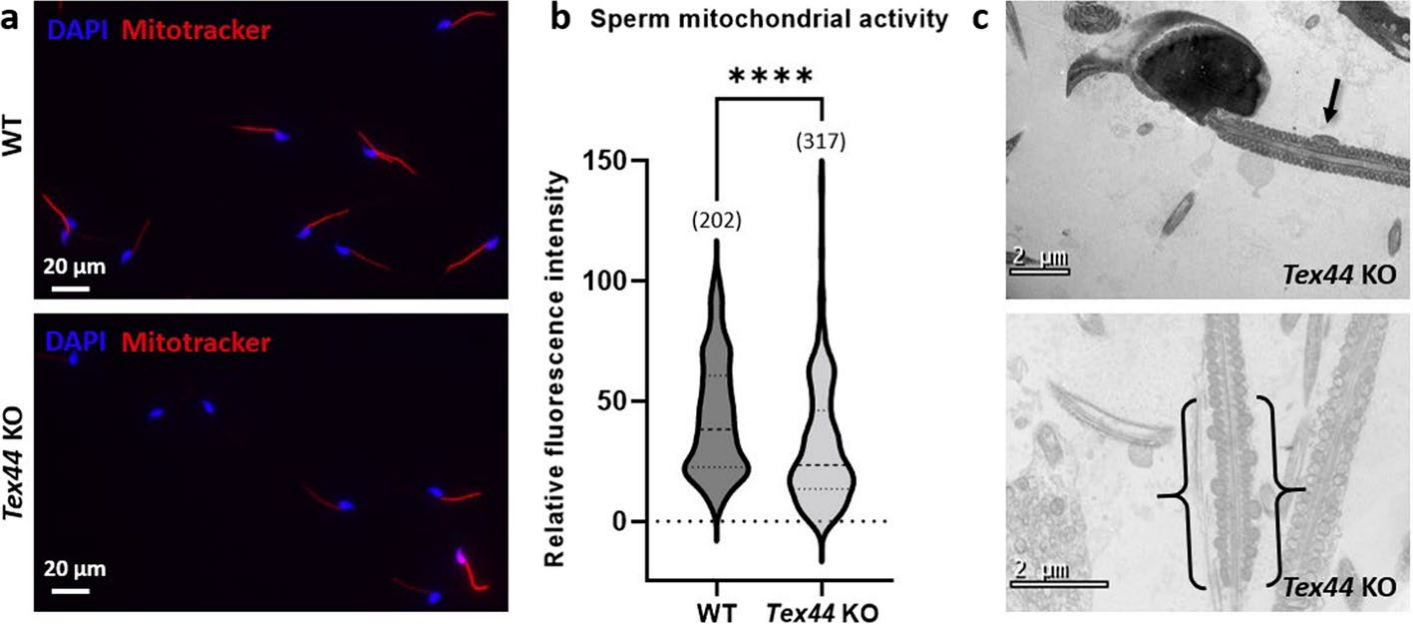
Characterization of the sperm mitochondrial activity by Mitotracker™ CMTMRos staining (**a**, **b**) and examples of mitochondrial abnormalities observed in the *Tex44*-KO sperm (**c**). **a** Mitotracker CMTMRos representative stainings on WT and *Tex44*-KO sperm and analysis of the relative fluorescence intensity (**b**). *Tex44*-KO sperm show a lower mitochondrial activity. Numbers between brackets indicate the number of sperm analyzed. **** *p* < 0.0001. (**c** Electron microscopy analysis of longitudinal sections of *Tex44*-KO flagellum sperm. Example of abnormal size of mitochondria is shown between brackets and supernumerary mitochondria shown by an arrow

Given the major defect in sperm motility, we used Mitotracker**™** CMTMRos, a probe that passively diffuses across the plasma membrane and accumulates in active mitochondria, to characterize the mitochondrial activity of WT and *Tex44*-KO sperm (Fig. 9). As presented in the violin plot (Fig. 9b), we noticed that *Tex44*-KO sperm statistically showed a lower fluorescence intensity compared to the WT sperm which are more uniformly distributed between low and high relative fluorescence intensities (Fig. 9a). These results suggest that *Tex44*-KO sperm show a significantly lower mitochondrial activity than the WT sperm. In some cases, abnormal mitochondrial structures can be observed in electron microscopy (Fig. 9c). Even if this phenotype was never observed in WT, it remained rare in KOs preventing any statistical comparison.

### Expression and localization of TEX44 on the human sperm

**Fig. 10:**
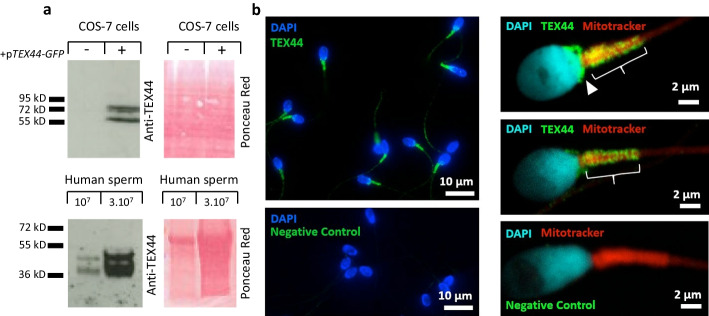
Expression of TEX44 in human sperm. **a** Western blot with extracts of COS-7 cells transfected or not with human TEX44-GFP and of human sperm, revealed by a polyclonal anti-TEX44 antibody. The Ponceau Red staining is provided as a loading control. Classical green immunofluorescence (**b**) and super-resolution STED green fluorescence (**c**) showing the main localization of TEX44 on the midpiece of human sperm flagellum (shown by a curly bracket) and on the post-acrosomal part of the sperm head (shown by an arrowhead) at the same time as the labeling of mitochondria by a Mitotracker**™** probe (red fluorescence)

Unfortunately, no specific antibodies against the mouse TEX44 protein are commercially available preventing us from seeking its localization. We nevertheless could study the expression and localization of TEX44 in human sperm. A polyclonal anti-TEX44 has been validated by Western blot (Fig. 10a). Briefly, this antibody recognized the human form of TEX44 coupled with GFP in transfected COS-7 cells with an expected ~ 69 kDa band (42 kDa for human TEX44 + 27 kDa for the GFP). We then used it on human sperm extracts and detected the expression of TEX44 with an expected ~ 42 kDa band (Fig. 10a). It should be noted that, at least, a double band has been detected in both conditions suggesting a cleavage or post-translational modification of the protein since *TEX44* is a mono-exonic gene. The same antibody validated in Western blot was used to localize TEX44 in human sperm by immunofluorescence (Fig. 10b) and by super-resolution STED fluorescence (Fig. 10c). Following both approaches, we observed specific staining on the midpiece of the flagellum accompanied or not by post-acrosomal staining of the sperm head. The staining appears as a network surrounding mitochondrial helixes.

### Bioinformatics analysis on TEX44 structure and function.

Given the potential colocalization between TEX44 and mitochondria, we used some bioinformatic tools to further characterize the structure and function of this protein. We used InterPro (https://www.ebi.ac.uk/interpro/) to predict its potential domains, but the prediction did not show any result other than “Domain of unknown significance” (DUF4678**)**. As there is no crystal structure for TEX44, we used the Alphafold database (https://alphafold.ebi.ac.uk/) to retrieve the prediction for this protein structure (AF-Q53QW1-F1). The confidence scores for most amino acids are very low, with most regions being predicted as disordered (Supplementary Fig. 2a). This was further validated using the IUPred3 tool [15] (https://iupred.elte.hu/plot) and the PDBSum tool (https://www.ebi.ac.uk/thornton-srv/databases/pdbsum/) (Supplementary Fig. 2b). Since we noticed the presence of several alpha helices, we used the DeepTMHMM tool (https://dtu.biolib.com/DeepTMHMM) to interrogate whether TEX44 could be a membrane protein. The prediction suggested that there is no membrane domain (Supplementary Fig. 2c). We also used the STRING database [16] (https://string-db.org/) to explore potential protein interactors of TEX44 (Supplementary Fig. 2d). The figure shows the network with an interaction score of 0.150 (low confidence), however, 3 out of 4 interactors experimentally determined (CPT1B, TIMM17B, and MICOS13) are still present if we use a higher interaction score (0.40; medium confidence). Notably, these proteins are involved in mitochondrial function.

## Discussion

Among the list of candidate genes within the *Mafq1* QTL interval, responsible for the hypofertility phenotype observed in the Rc3 IRCS strain [7], the *Spata3* gene appeared to be a strong candidate gene. Its deletion partially reproduced the phenotype of the hypofertile IRCS strain. Namely, it induced morphological alterations in sperm, including a defective anchoring of the acrosome to the nucleus and the persistence of residual bodies in the sperm midpiece, as well as hypofertility only observed in vitro [8]. However, in one of the latest versions of the mouse and human genomes, a new gene, *Tex44*, has been described in this interval of interest on mouse chromosome 1 and its syntenic region on human chromosome 2. Its function is unknown, but *Tex44* is the only other candidate gene that is specifically expressed in the testis (at the spermatid stage) and shows genetic polymorphisms in the hypofertile IRCS mouse line, predicted to affect protein function as listed in Supplementary Table 1. *Tex44* presents with a longer list of missense SNPs than *Spata3* but analysis software predicts that the ones in *Tex44* have lower impacts on protein function. Interestingly, we found that both *Spata3* and *Tex44* are exclusively present in placental mammals, as already observed for genes expressed at the latest spermatogenic stages [17] and correlated to the rapid evolution of genes responsible for male reproductive fitness.

In order to study the role of the TEX44 protein in male fertility particularly in spermatogenesis, we generated *Tex44*-KO mice. In vivo crossings with KO males showed a reduced average litter size. Though some WT females were positive for a vaginal plug after mating with KO males, no birth could be detected after 3 weeks. In order to understand if this in vivo hypofertility was either due to an embryonic development defect or to a fertilization defect, we achieved in vivo fertilization assays with WT superovulated females that were mated overnight with WT or *Tex44*-KO males. The next day, oocytes of females showing a vaginal plug were retrieved and the in vivo fertilization rate appeared to be drastically reduced in the KO group, confirming a fertilization defect of the *Tex44*-KO sperm. In a similar way, *Tex44*-KO sperm almost completely failed to fertilize WT oocytes both in cumulus-intact IVF and in zona-free IVF assays. Interestingly, increasing the sperm concentration of *Tex44*-KO by tenfold during zona-free IVF allowed the restoration of the fertilization index to the same level as the control. The deletion of *Tex44* thus shows a more drastic phenotype of hypofertility than the original Rc3 line.

Even though *Tex44*-KO males showed no abnormalities in their testicular histology and their epididymal sperm count, evoking normal spermatogenesis, their sperm showed a drastic reduction of motility and important ultrastructural anomalies. In fact, the progression of the *Tex44*-KO sperm through the different stages of maturation in the epididymis was correlated to a progressive deterioration of the morphological phenotype. This latter evolved from a majority of midpiece-principal piece (MP-PP) disjunctions in the caput epididymis to a majority of severe bending of the flagellum in the cauda epididymis. This could be explained by the fact that during their transit through the epididymis, sperm gradually acquire their motility [18–20]. Since *Tex44*-KO sperm present a weakness at the MP-PP junction, the initial acquisition of their mobility leads to a break at this specific region (severe hairpin bending).

The drastic reduction of *Tex44-*KO sperm motility is a direct consequence of important morphological sperm abnormalities. Interestingly, the low percentage (< 10%) of sperm that do not present morphological abnormalities is nearly the same as the one of motile sperm. These few “normal” sperm could explain the fact that the *Tex44*-KO males are severely hypofertile but could still rarely sire in vivo and in vitro. This hypothesis has been validated by the restoration of the fertilization index in vitro to a control value when the *Tex44*-KO sperm concentration was increased tenfold.

Most of the abnormalities observed on the *Tex44*-KO sperm have already been described previously in the literature and can potentially help us in the comprehension of TEX44's function in mice. They can define the phenotype as terato-asthenozoospermia and are reminiscent of one of the anomalies observed in the MMAF (multiple morphological anomalies of the flagellum) syndrome, a mosaic of defects in sperm flagellum morphology leading to reduced sperm motility [21].

For example, the absence of SEPTIN4 [14] or TAT1 [22], which are critical components of the sperm annulus, leads to males that are infertile in knock-out mouse models. These KO mice show a complete lack of sperm motility and structurally defective sperm with MP-PP disjunction and hairpin bending of the tail, disruption of the axial structures, absence of annulus, and abnormal mitochondrial sheath structure. The mouse knockout of *Cabs1,* a gene coding for a spermatid-specific calcium-binding protein, leads to male subfertility with a disorganization of the MP-PP junction and an abnormal annulus.

The absence of SEPP1, a crucial component of the selenium dietary micronutrient delivery pathway in male germ cells development, shows the same defective phenotype as in the absence of SEPTIN4 and TAT1, but with the maintenance of an annulus [23].

Recently, the protein LRRC46, a member of the Leucine-rich repeat protein (LRRC) family, has been described to play a role in the mouse sperm flagellum biogenesis. Its location is restricted to the midpiece, like TEX44. However, the knockout of *Lrrc46* showed typical MMAF phenotypes including coiled, short, and irregular flagella [24]. The *Tex44*-KO doesn’t seem to affect deeply the annulus as SEPTIN4 is normally detected at the right position and, in electron microscopy images, the expected triangular dense structure of the annulus is visible between the midpiece and principal piece, in spite of surrounding abnormalities.

Damage in the integrity of microtubule doublet structure has been shown to be associated with sperm motility defects and male infertility, as seen for the deletion of *Ccdc176* [25], *Tmem232* [26], *Cfap97d1* [27], *Dnah17* [28], *Trll9* [29], *Pla2g3* [30] and *Vdac3* [31] in mice. Missing microtubule doublets have also frequently been observed in the principal piece of *Tex44*-KO sperm. The intra-flagellar traffic is apparently affected. It is not clear whether this anomaly is a primary result of the absence of TEX44 expression or if it is a secondary consequence of the disjunction and break taking place between the midpiece and the principal piece, preventing any protein movement downstream of the annulus.

In the literature, RABL2, a member of the RAS GTPase superfamily, has been described to play a role in the mouse sperm intra-flagellar transport and the tail assembly [32]. Its location is restricted to the midpiece, like TEX44. However, a mutation in a critical protein–protein interaction domain in a mouse line model resulted in male sterility with decreased sperm output and severe motility defects but with normal sperm morphology.

Knowing that no specific antibodies against TEX44 are available in mice we however had a look at the human protein expression and localization. After validating an antibody in Western blot in COS-7 transfected cells, we could confirm TEX44 expression in human sperm extracts. In order to localize the protein, we performed some immunofluorescence assays by classical and super-resolution (STED) approaches. TEX44 appeared to be localized on the post-acrosomal region of the sperm head and the midpiece of the sperm flagellum. In fact, TEX44 seems to encompass the mitochondrial sheath of the midpiece in a spiral-like conformation. Interestingly, looking at the STRING database (https://string-db.org/), TEX44 is described as interacting with human TIMM17B, CPT1B [33, 34], and MICOS13 proteins (Detected by validated two-hybrid assay). These three proteins are respectively expressed in the inner, the outer, and the cristae mitochondrial membrane. However, it is not clear if TEX44 is a mitochondrial protein (and in which section of the organite) or if it is surrounding. Imaging by super-resolution rather suggested a structural role around the midpiece mitochondrial sheath. The decrease of mitochondrial activity observed in the *Tex44*-KO and detected by the MitoTracker probe could rather be an indirect consequence of an abnormal setup of the mitochondrial network. It is interesting to notice that the CABS1 protein mentioned above has been detected in the mitochondrial inner membrane in rat sperm [35]. The KO of the *Gk2* and *Gykl1* genes, coding for mitochondrial proteins specifically expressed in spermatids, show some similarities of phenotype: flagellum bending and some disorganization of the mitochondrial sheath [36]. The mitochondrial disorganization that we observed could be due to the absence of TEX44 if this protein is necessary to structure the mitochondrial sheath. It could also be a consequence of the perturbations that affect trans-flagellar traffic and that compromise mitochondrial maintenance. Mitochondrial dysfunction could worsen sperm mobility already jeopardized by flagellum breaking.

Though our interest in the *Tex44* gene was motivated by its location within the *Mafq1* interval, we consider that *Tex44-KO* males share few phenotypic traits with the original Rc3 IRCS line as summarized in Supplementary Table 4. Flagellar defects would have been observed in Rc3 males if TEX44 function was affected. Both *Spata3* and *Tex44* genes seem to have independent functions as the defects observed in both KO target different subcellular compartments and also independent regulations as both genes are above 400 kb distant and as *Spata3* is correctly expressed in *Tex44*-KO testis and reciprocally (Fig. 2b). Nevertheless, this study allowed us to reveal a new gene important for sperm structure and function.

## Conclusion

Even though the knockout of *Tex44* does not really reproduce the phenotype observed in the Rc3 IRCS males (moderate hypofertility, abnormal anchoring of the acrosome to the nucleus, and persistence of residual bodies at the sperm midpiece) [7], it exhibits other alterations (flagellum defects, abnormal mitochondria). Here, we described a new gene, *Tex44*, that is implicated in a reproductive phenotype in mice. Taken together, our results show that *Tex44* in mice is implicated in the correct set-up of the sperm flagellum during spermatogenesis and more precisely during spermiogenesis. The precise function of the TEX44 protein, its interaction with other sperm proteins, as well as its involvement in humans remain to be investigated.

### Supplementary Information

Supplementary Material 1: Figure 1. Evolutionary history and expression of *Spata3* compared to *Tex44*. (a). The ensemble Gene tree for *Spata3* shows that the gene first appeared in placental mammals. (b). Single-cell RNA sequencing data confirmed the tissue-specific expression of *Spata3* in early and mainly late spermatids, just like *Tex44*. (c). Data from The Huma Protein Atlas shows that the expression of *Tex44* and *Spata3* is similar in different male germ cell types.

Supplementary Material 2: Figure 2. Bioinformatics analysis on TEX44 structure and function. (a). The Alphafold prediction for TEX44 shows a disordered protein with very low confidence scores. (b). The IUPred3 and PDBSum tools confirm an overall disordered protein with some scarce alpha helices. (c). DeepTMHMM prediction suggests that TEX44 is not a membrane protein. (d). The STRING network for TEX44 displays four main clusters of interactors, with four experimentally determined interactors involved in mitochondrial function.

Supplementary Material 3: Table 1. List of SNPs observed in the *Tex44* and *Spata3* genes of the *Spretus* species compared to the C57BL/6 reference genome and their potential impact according to the Polyphen-2 and SIFT softwares.

Supplementary Material 4: Table 2. List of primers used, their sequence, position, expected amplicon size (bp) and use.

Supplementary Material 5: Table 3. List of placental mammals not reported by Ensemble.

Supplementary Material 6: Table 4. Comparison of the phenotypes of the Rc3 IRCS line, the *Tex44*-KO and the *Spata3*-KO lines.

Supplementary Material 7: Table 5. Raw data corresponding to Figures 3b,c,d, 4b and 7b.

## Data Availability

Not applicable.

## Abbreviations

IRCS: Interspecific Recombinant Congenic Strain
QTL: Quantitative Trait Locus
CRISPR: Clustered Regularly Interspaced Short Palindromic Repeats
TEX44: Testis-expressed protein 44
WT: Wild-type
MMAF: Multiple Morphological Abnormalities of the Flagella
STED: Stimulated emission depletion
FR: Fertilization Rate: Average number of fertilized oocytes among the total number of oocytes
FI: Fertilization Index: Average number of fused sperm per oocyte
MP: Midpiece
PP: Principal piece
