# Supplementary figures and images for: The lack of Tex44 causes severe subfertility with flagellar abnormalities in male mice
Source: Cell Mol Biol Lett. 2024 May 15;29:74. doi: 10.1186/s11658-024-00587-5 (PMC11094962; doi:10.1186/s11658-024-00587-5)

## Slide 1
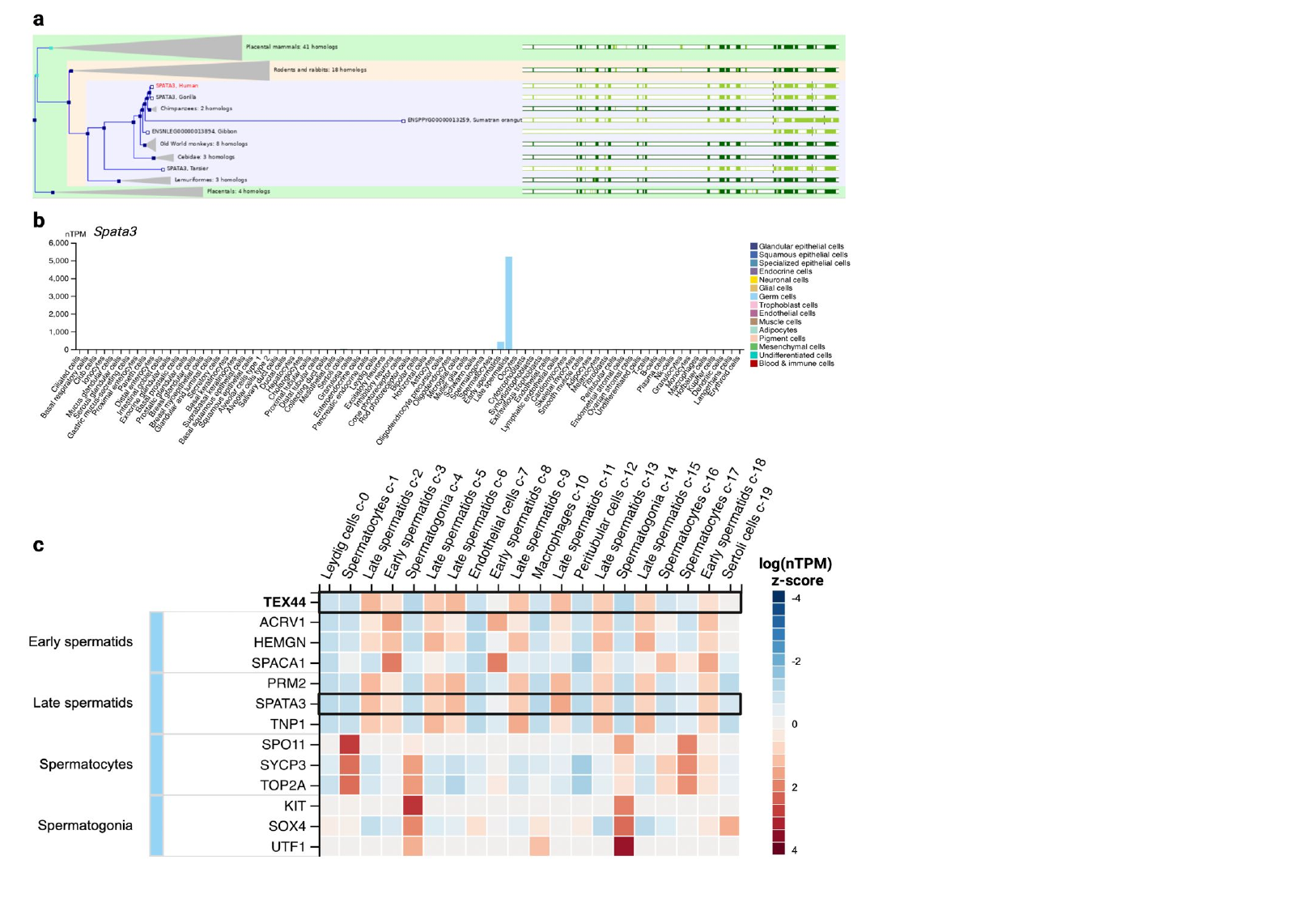

Supplement: Supplementary file 1 — Supplementary Material 1: Figure 1. Evolutionary history and expression of Spata3 compared to Tex44. (a). The ensemble Gene tree for Spata3 shows that the gene first appeared in placental mammals. (b). Single-cell RNA sequencing data confirmed the tissue-specific expression of Spata3 in early and mainly late spermatids, just like Tex44. (c). Data from The Huma Protein Atlas shows that the expression of Tex44 and Spata3 is similar in different male germ cell types. [file 11658_2024_587_MOESM1_ESM.pptx]

## Slide 1
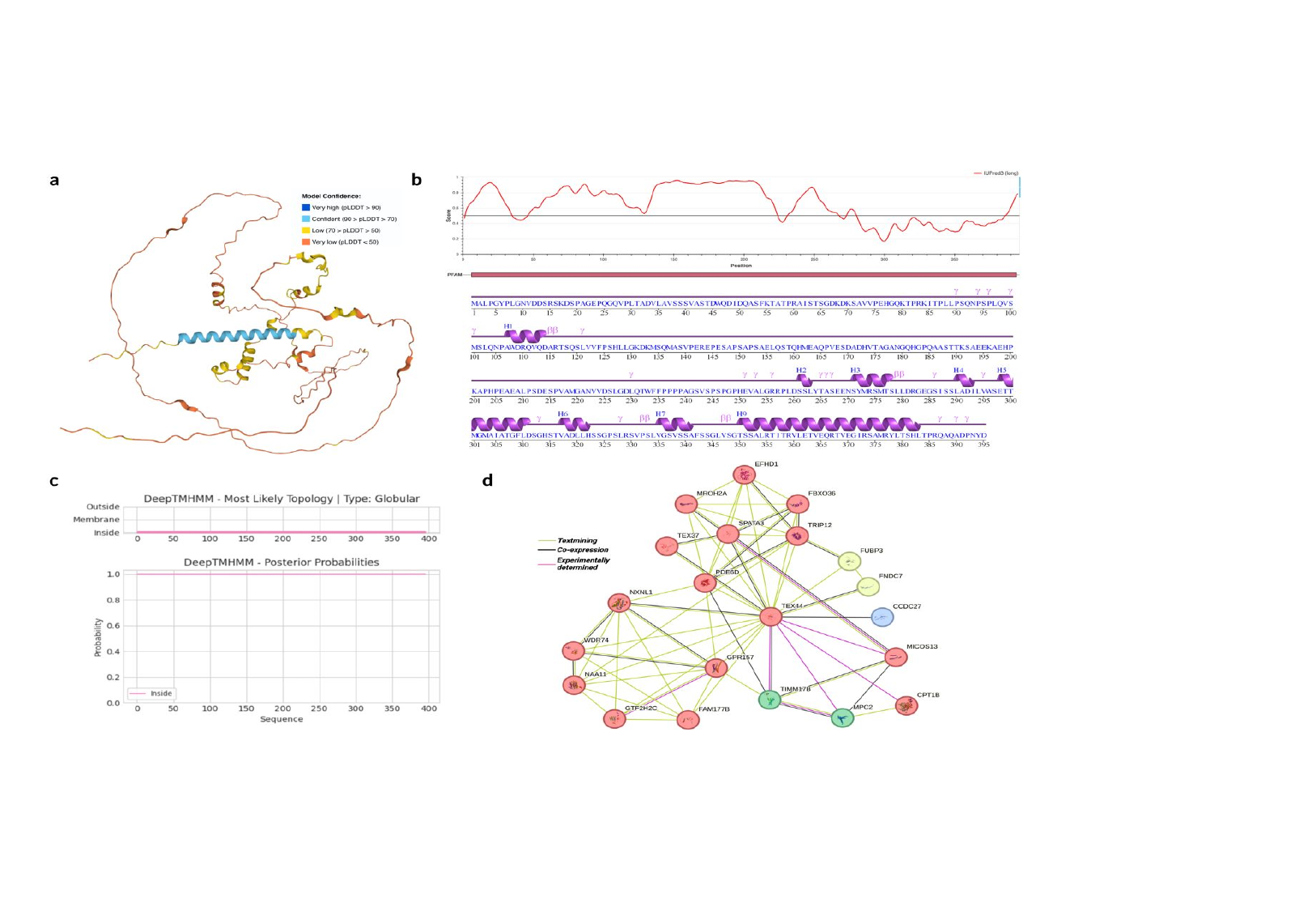

Supplement: Supplementary file 2 — Supplementary Material 2: Figure 2. Bioinformatics analysis on TEX44 structure and function. (a). The Alphafold prediction for TEX44 shows a disordered protein with very low confidence scores. (b). The IUPred3 and PDBSum tools confirm an overall disordered protein with some scarce alpha helices. (c). DeepTMHMM prediction suggests that TEX44 is not a membrane protein. (d). The STRING network for TEX44 displays four main clusters of interactors, with four experimentally determined interactors involved in mitochondrial function. [file 11658_2024_587_MOESM2_ESM.pptx]

## Slide 1
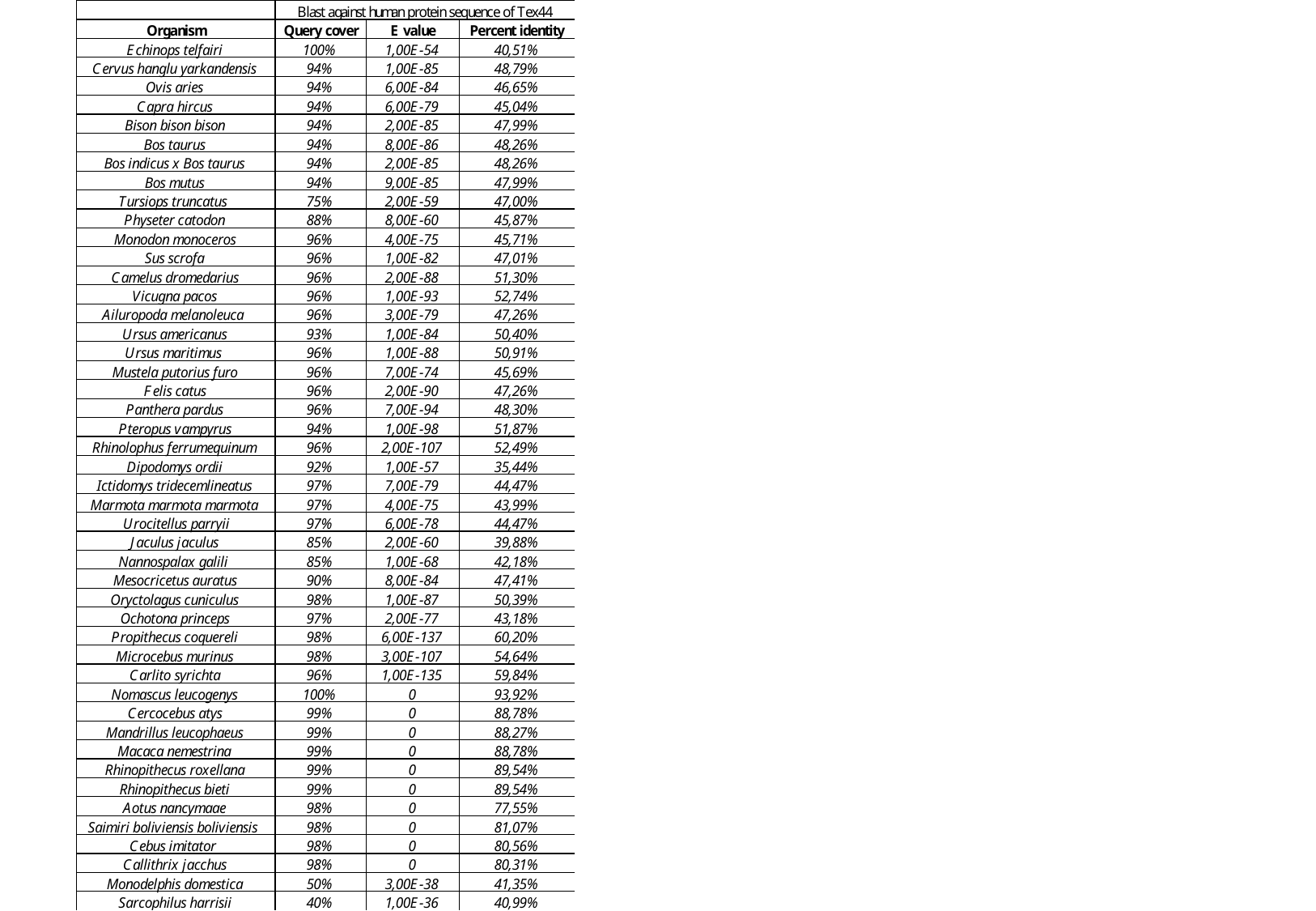

Supplement: Supplementary file 5 — Supplementary Material 5: Table 3. List of placental mammals not reported by Ensemble. [file 11658_2024_587_MOESM5_ESM.pptx]
